# Eradication of Cancer Cells Using Doxifluridine and Mesenchymal Stem Cells Expressing Thymidine Phosphorylase

**DOI:** 10.3390/bioengineering11121194

**Published:** 2024-11-26

**Authors:** Xutu Wang, Ian Peng, Ching-An Peng

**Affiliations:** 1 Voiland School of Chemical Engineering and Bioengineering, Washington State University, Pullman, WA 99164, USA; 2 Department of Bioengineering, University of Pennsylvania, Philadelphia, PA 19104, USA; 3 Department of Chemical and Biological Engineering, University of Idaho, Moscow, ID 83844, USA

**Keywords:** gene-directed enzyme prodrug therapy, mesenchymal stem cell, lentiviral vector, thymidine phosphorylase, doxifluridine, 5-fluorouracil

## Abstract

Gene-directed enzyme prodrug therapy (GDEPT) has been developed over several decades as a targeted cancer treatment aimed at minimizing toxicity to healthy cells. This approach involves three key components: a non-toxic prodrug, a gene encoding an enzyme that converts the prodrug into an active chemotherapy drug, and a gene carrier to target cancer cells. In this study, the prodrug doxifluridine was enzymatically converted into the chemotherapy drug 5-fluorouracil via thymidine phosphorylase, using human mesenchymal stem cells (hMSCs) as delivery vehicles. The hMSCs were first transduced with thymidine phosphorylase-encoded lentiviral vectors produced by HEK293T cells, then co-cultured with A549 adenocarcinoma cells in the presence of doxifluridine. The results showed that after 3 days of prodrug treatment, cell viability in both A549 cancer cells and hMSCs dropped by about 50%, and by day 5, viability had decreased to 10%. In summary, exogenous thymidine phosphorylase expressed in hMSCs successfully converted the non-toxic prodrug doxifluridine into the chemotherapy agent 5-fluorouracil, effectively eliminating both cancer cells and hMSCs within a short period.

## 1. Introduction

Gene-directed enzyme prodrug therapy (GDEPT) has been extensively developed over the years [1]. In this approach, an inactive prodrug is converted into an active chemotherapy drug by an enzyme that is specifically expressed in cancer cells [2,3]. This localized enzymatic conversion ensures that the chemotherapy drug is mainly produced at the tumor site, minimizing the side effects that would typically occur with systemic circulation of the drug [4]. GDEPT or suicide gene therapy consists of three main components: (1) a chemotherapy drug and its corresponding inactive prodrug, (2) a transfer gene encoding the enzyme that catalyzes the prodrug conversion, and (3) a carrier system to deliver the gene to cancer cells [5]. To initiate the process, a transfer gene that encodes the required enzyme is delivered to the cancer cells using a suitable carrier, enhancing the enzyme expression and the prodrug-to-drug conversion rate [6].

GDEPT allows for greater flexibility and precision in cancer treatment, with various prodrug–enzyme pairs available. One notable example is the conversion of the prodrug doxifluridine into the chemotherapy drug fluorouracil (5-FU) by the enzyme thymidine phosphorylase (TP) [7], a nucleoside metabolism enzyme with two identical subunits for thymidine binding [8]. TP, located on chromosome 22q13, forms a dimer with a molecular mass of 102 kDa and plays a key role in the pyrimidine salvage pathway by catalyzing the conversion of thymidine to thymine [9,10]. TP is overexpressed in several cancer types, including gastric [11], breast [7], and colorectal cancers [12]. It has been shown to promote various cancer-related processes, such as tumor angiogenesis, invasion, metastasis, immune evasion, and resistance to apoptosis [13]. Given its elevated presence in cancer cells, TP has become a valuable target in cancer therapy, particularly in facilitating the conversion of doxifluridine to 5-FU [8,9].

In this study, the transfer gene for TP was delivered to human mesenchymal stem cells (hMSCs) using both non-viral and viral vectors to facilitate prodrug conversion. Lentivirus is particularly effective in transfecting long DNA sequences into non-dividing cells, offering superior efficiency compared to other viral vectors [14]. Additionally, lentiviral vectors are regarded as safe gene delivery vehicles because the modifications made to their viral genes do not continue replicating after transfection into the target cell [15,16]. Lentiviral vectors also induce minimal inflammation due to safety modifications made to the surface proteins responsible for binding target cells [17]. Here, lentivirus was used to deliver TP genes into hMSCs. When tested against the non-viral vector polyethyleneimine (PEI) [18], lentivirus demonstrated higher gene delivery efficiency in hMSCs without causing cellular toxicity.

Since the level of gene expression and activity directly influences the rate of prodrug-to-drug conversion and the overall therapeutic efficacy [19,20], optimizing the gene delivery system in GDEPT is crucial, particularly when selecting a suitable carrier. In this study, hMSCs were chosen to deliver the TP gene to cancer cells due to their tumor-tropic nature and low immunogenicity [21,22]. MSCs possess a natural homing ability via specific receptors that interact with cytokines secreted by cancer cells, such as TNF-α [23]. Once MSCs carrying the TP gene home to tumor sites, the prodrug doxifluridine can be locally converted to the chemotherapy drug 5-FU by the TP enzyme. Unlike other gene carriers, such as viruses, MSCs provoke minimal immune responses, enhancing the safety profile of this therapeutic approach [21,22].

## 2. Materials and Methods

### 2.1. Construction of TP-Encoded Plasmid

The cDNA of thymidine phosphorylase (TP) was cloned from the pcDNA3.1(+)-C-eGFP-TP plasmid (obtained from GenScript, Piscataway, NJ, USA) using polymerase chain reaction (PCR) with forward primer 5′-GCCATGGATATCATGGCAGCCTTGATGACCCC-3′ and reverse primer 5′-GATCTCGAATTCTTGCTGCGGCGGCAGAACG-3′. The TP PCR amplification was performed using high-fidelity Phusion polymerase (New England BioLabs [NEB], Ipswich, MA, USA) in a T-100 thermocycler (Bio-Rad, Hercules, CA, USA) with the following cycling conditions: initial denaturation at 98 °C for 30 s, followed by 35 cycles of denaturation at 98 °C for 10 s, annealing at 66°C for 30 s, and extension at 72 °C for 15 s. A final extension was conducted at 72 °C for 5 min. The resulting TP PCR product was purified using the Monarch PCR & DNA cleanup kit (NEB) and verified by 1% agarose gel electrophoresis. The pHR-SFFV-GFP plasmid (Addgene #80409, Watertown, MA, USA) and the TP PCR product (insert) were both digested with the BamHI restriction enzyme (NEB). The digested plasmid and TP insert were ligated using T4 DNA ligase (NEB) to form the pHR-SFFV-TP-GFP plasmid. This plasmid was further amplified by transforming NEB5α-competent *E. coli* cells (NEB) and purified using a Plasmid Miniprep kit (QIAGEN, Germantown, MD, USA).

### 2.2. Lentiviral Production by Co-Transfection of HEK293T Cells

To produce lentiviral vectors encoding the TP-GFP gene, a 1 mg/mL solution of linear polyethylenimine (PEI, MW = 25 kDa; Sigma-Aldrich, St. Louis, MO, USA) was prepared and sterilized using a 0.22 µm filter. Quantities of 6 µg of each plasmid—pMD2.G (virus envelope plasmid; Addgene #12259, Watertown, MA, USA), pCMV-dR8.2 dvpr (packaging plasmid; Addgene #8455), and the constructed pHR-SFFV-TP-GFP plasmid—were mixed with the PEI solution at N/P ratios of 3, 5, and 7, respectively, in 100 µL of Dulbecco’s Modified Eagle’s Medium (DMEM; Thermo Fisher Scientific, Waltham, MA, USA). The PEI–plasmid complexes were incubated at room temperature in a biosafety hood for 30 min, then directly added to a T-75 flask containing human embryonic kidney HEK293T cells (ATCC, Manassas, VA, USA) at 40% confluence. These cells were cultured in DMEM supplemented with 1% penicillin–streptomycin (Thermo Fisher Scientific) and 10% fetal bovine serum (FBS; Thermo Fisher Scientific) and incubated at 37 °C with 5% CO_2_ in a humidified incubator.

Following co-transfection of the three plasmids using PEI, 10 mL of lentivirus-containing medium from HEK293T cells was harvested and replaced with fresh DMEM every 24 h for 3 days. The collected supernatants were filtered using a 0.45 µm syringe filter. To assess the expression of TP-GFP at the three different N/P ratios, the cells were imaged daily using fluorescent microscopy (DMi8 equipped with a Leica EC3 camera; Leica Microsystems, Wetzlar, Germany). Additionally, the relative fluorescent intensities were quantified using a spectrophotometer (SpectraMax M2e; Molecular Devices, Sunnyvale, CA, USA). On the third day, the culture supernatant containing lentiviral particles was concentrated as described in a previous study [24]. Briefly, the virus-containing supernatant was mixed with an equal volume (10 mL) of Polybrene solution (320 µg/mL) and incubated at 37 °C for 30 min. The mixture was then centrifuged at 10,000× *g* for 10 min at 4 °C. The resulting lentivirus pellet was resuspended in 1 mL of phosphate-buffered saline (PBS) and stored at −80 °C for later use.

### 2.3. TP-GFP Protein Expression in hMSCs After Viral and Non-Viral Transfection

A total of 1 × 10^5^ human mesenchymal stem cells (hMSCs) (Lonza, Alpharetta, GA, USA) were seeded into 6-well culture plates, with each well containing 1 mL of alpha MEM Essential Medium (α-MEM, Thermo Fisher Scientific) supplemented with 1% penicillin/streptomycin, 10% FBS, and 1% L-glutamine (Thermo Fisher Scientific). After overnight incubation, the hMSCs were infected with 100 µL of concentrated lentiviral vectors harvested 72 h post-transfection of HEK293T cells, using three plasmids complexed with PEI at an N/P ratio of 5. TP-GFP protein expression was monitored and imaged using a fluorescence microscope at 36 and 48 h after viral infection. Additionally, 48 h post-viral infection, hMSCs were trypsinized and re-suspended in α-MEM medium. The cells were then loaded into a flow cytometer cell sorting machine (SH 800; Sony, New York, NY, USA) for detection of green fluorescence. The percentage of GFP-positive cells was calculated by counting the number of cells using a Leica DMi8 microscope under bright-field and fluorescent view, respectively. Following the sorting process, hMSCs expressing TP-GFP protein were collected and cultured for subsequent GDEPT experiments. For comparison, 100 µL of a non-viral PEI/plasmid pHR-SFFV-TP-GFP mixture (prepared at N/P ratios of 5 and 7, respectively) was incubated at room temperature for 30 min and then added to 6-well culture plates containing 1 × 10^5^ hMSCs. The cells were cultured overnight, and TP-GFP expression was evaluated and imaged via fluorescence microscopy 48 h after non-viral transfection. The relative fluorescent intensities were further quantified using a spectrophotometer (SpectraMax M2e; Molecular Devices).

### 2.4. Protein Extraction and Enzyme Activity Assay

Human MSCs expressing TP-GFP were harvested using 0.25% trypsin, then centrifuged at 400× *g* for 5 min to form a cell pellet. The pellet was resuspended in 100 µL of RIPA buffer (Thermo Fisher Scientific) containing 1 µL of protease inhibitor and incubated on ice for 15 min to lyse the cells and release TP-GFP protein along with other cell components. The cell suspension was sonicated on ice in three 10 s bursts, with 30 s intervals between each burst, and then centrifuged at 14,000× *g* for 15 min. The supernatant containing crude proteins was transferred to a new tube, while the pellet was discarded. Next, an equal volume of potassium phosphate buffer (pH 7.4) was added to the supernatant. To this mixture, 10 µL of 200 M doxifluridine was added and incubated on ice for 5 min. An initial absorbance reading of the sample was taken using a spectrophotometer (SpectraMax M2e; Molecular Devices) to establish a baseline. The sample was then incubated at 37 °C with gentle shaking at 70 rpm for 1 h. After incubation, 10 µL of 2 M NaOH was added to stop the reaction, and the sample was placed on ice for 5 min. The absorbance of each sample was measured again, and the difference in absorbance at around 305 nm (the absorbance peak of 5-FU) was used to calculate TP enzyme activity. The calculations were based on a calibration curve and the results of a BCA assay (Thermo Fisher Scientific) for each sample.

### 2.5. SDS-PAGE Gel and Western Blot Analysis

The crude protein extract was mixed with a 2× Laemmli sample buffer (Bio-Rad) containing 5% β-mercaptoethanol (Thermo Fisher Scientific) at a 1:1 ratio, and the mixture was thoroughly pipetted to ensure proper mixing. The sample was then heated at 95 °C for 5 min in a water bath to denature the proteins. Both the protein mixture and a protein ladder (Bio-Rad) were loaded onto a 12% SDS-PAGE gel, prepared with 1× Tris/glycine/SDS buffer (Bio-Rad), and electrophoresed for 1 h at 150 V. Immediately after electrophoresis, the proteins were transferred onto a nitrocellulose membrane (Bio-Rad) using a Trans-Blot Semi-Dry Transfer System (Bio-Rad) at 20 V for 1 h. The membrane was washed three times in Tris-buffered saline containing 0.1% Tween-20 (TBST) for 5 min per wash. It was then blocked in a 5% milk powder blocking buffer for 1.5 h with gentle shaking at room temperature. The membrane was incubated overnight at 4 °C with the anti-TP primary antibody (GenScript) diluted in a blocking buffer at a ratio of 1:1000 while being continuously shaken. Unbound primary antibody was washed away with three TBST washes. Next, the membrane was incubated for 1 h at room temperature with a secondary antibody (anti-mouse IgG HRP-conjugated; Sigma) diluted 1:1500, followed by three more TBST washes. The membrane was then treated with a mixture of ECL A and B solutions (Thermo Fisher Scientific) for 1 min and subsequently visualized using a chemiluminescence imager (Azure 280; Azure Biosystem Inc., Dublin, CA, USA).

### 2.6. Co-Culture of Transfected hMSCs and A549 Cells with Doxifluridine Treatment

Human mesenchymal stem cells (hMSCs) expressing TP-GFP protein (1 × 10^5^ cells) were co-cultured with A549 cells at three different inoculation ratios: 2:1, 1:1, and 1:2. The number of hMSCs expressing TP-GFP protein was maintained at 1 × 10^5^, while the number of A549 cells was adjusted accordingly to achieve the respective inoculation ratios. After the cells attached and spread overnight, 200 µM of prodrug doxifluridine was added to each co-culture well. Cell morphology was monitored and imaged over a period of 5 days, and cell viability was assessed using the trypan blue exclusion method. For comparison, hMSCs, A549 cells, and co-culture of hMSCs and A549 cells (at a ratio of 1:1) were separately cultured and treated with 200 µM doxifluridine over 5 days. Additionally, A549 cells were cultured and treated with 200 µM 5-FU for 5 days as another control group.

## 3. Results and Discussion

### 3.1. TP-Encoded Plasmid Verification

**Figure 1:**
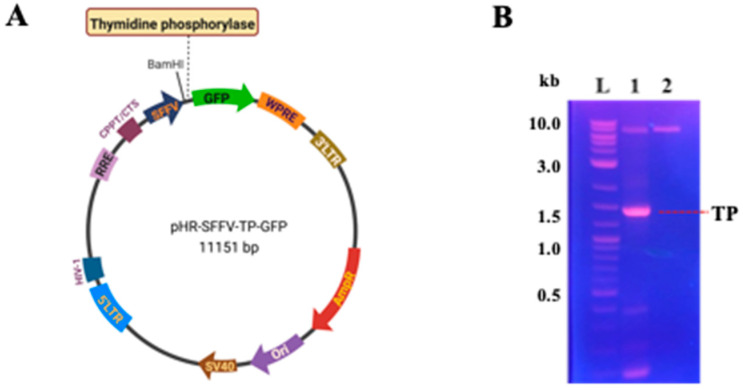
(**A**) Plasmid map of pHR-SFFV-TP-GFP, featuring the designed TP insert at the BamHI site upstream of the GFP sequence. (**B**) DNA electrophoresis gel image showing PCR cloning products of the TP sequence insertion. Lane L represents the DNA ladder; Lane 1 displays a band at 1.5 kb, indicating the successful cloning of the TP sequence from the constructed pHR-SFFV-TP-GFP plasmid; Lane 2 shows an empty band for the pHR-SFFV-GFP plasmid without the TP sequence insertion.

Figure 1A shows the construction of the TP-encoding recombinant plasmid (pHR-SFFV-TP-GFP), achieved by inserting the TP gene sequence (1500 bp) into the plasmid vector pHR-SFFV-GFP (9651 bp). The TP gene sequence from the pHR-SFFV-TP-GFP plasmid, amplified by PCR, was confirmed via DNA gel electrophoresis, showing a band of approximately 1.5 kb for TP (Figure 1B). The orientation of the inserted gene was checked using restriction enzyme analysis, and the open reading frames of the recombinant plasmid were verified by DNA sequencing.

### 3.2. Co-Transfection of HEK293T Cells for Lentiviral Production

**Figure 2:**
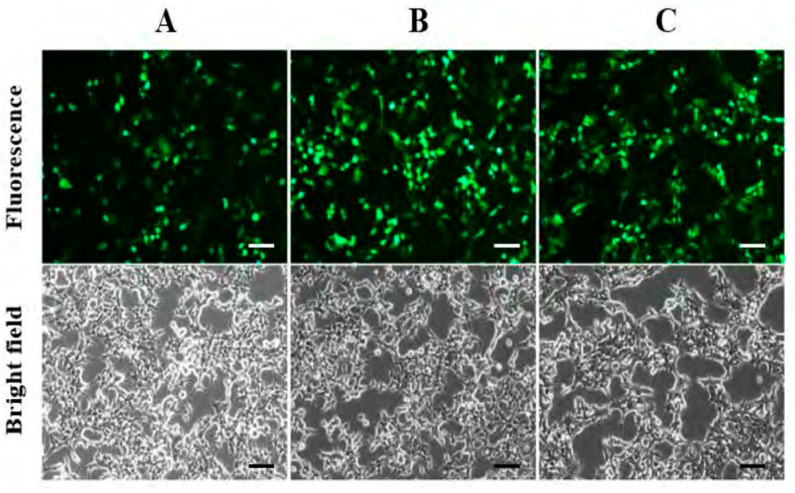
N/P ratio effect on co-transfection of HEK293T cells for lentiviral vector production. Fluorescence and bright-field images of TP-GFP protein expression in HEK293T cells after 72 h of co-transfection with plasmids to produce lentiviral vectors at varying N/P ratios of PEI/plasmid complexes: 3 (**A**), 5 (**B**), and 7 (**C**). The scale bar represents 100 µm.

Figure 2A–C display the TP-GFP protein expression levels in HEK293T cells co-transfected with three plasmids using cationic PEI at varying N/P ratios (PEI to plasmid DNA). As shown in Figure 2B, the N/P ratio of 5 resulted in the highest TP-GFP expression, which was slightly higher than the expression level observed with the N/P ratio = 7 (Figure 2C). At N/P ratio = 3, TP-GFP expression level was lower compared to the other two N/P ratios but still substantial (Figure 2A). This was verified by the relative fluorescent intensities measured by the spectrophotometer with 0.52 ± 0.09 (for N/P = 3), 1.00 ± 0.07 (for N/P = 5), and 0.86 ± 0.11 (for N/P = 7), respectively. The cell growth rates across these three groups were consistent with control cells (i.e., non-treated), and the cells appeared healthy throughout the co-transfection and TP-GFP expression period.

### 3.3. TP-GFP Expression in hMSCs by Non-Viral and Lentiviral Vectors

**Figure 3:**
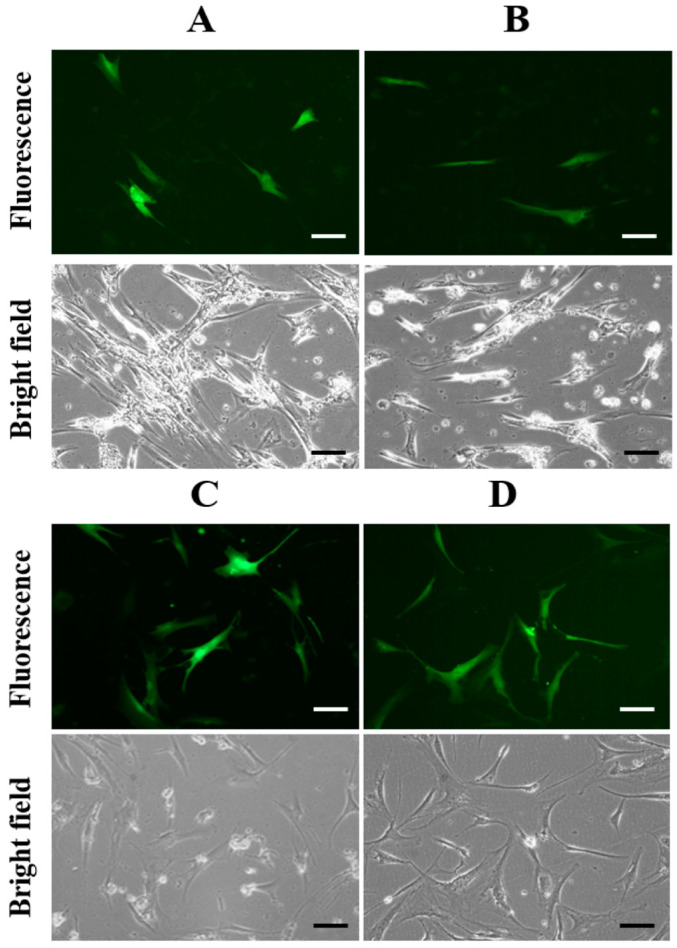
Comparison of TP-GFP expression in hMSCs using non-viral and viral transfection. Fluorescence and bright-field images of TP-GFP protein expression in human MSCs transfected with PEI/pHR-SFFV-TP-GFP complexes at N/P ratios of 5 (**A**) and 7 (**B**) for 48 h. Also shown are images taken at 36 h (**C**) and 48 h (**D**) after transfection with lentiviral vectors collected from the culture medium of HEK293T cells following 72 h co-transfection of PEI complexed with three plasmid (N/P ratio = 5). The scale bar represents 100 µm.

Figure 3 illustrates the levels of TP-GFP protein expression in hMSCs using both non-viral and viral vectors. Figure 3A,B show TP-GFP expression in hMSCs transfected with PEI/pHR-SFFV-TP-GFP complexes at N/P ratios of 5 and 7. The relative fluorescent intensities measured by the spectrophotometer were 0.24 ± 0.08 (for N/P = 5) and 0.12 ± 0.03 (for N/P = 7), respectively. The N/P ratio = 3 was excluded due to its relatively low expression, as seen in Figure 2A. Figure 3C (relative fluorescent intensity: 0.72 ± 0.11) and 3D (relative fluorescent intensity: 1.00 ± 0.09) depict TP-GFP expression in hMSCs 36 and 48 h post-infection with lentiviral vectors collected from the culture medium of HEK293T cells 72 h after co-transfection with PEI/plasmid complexes (N/P ratio = 5). Based on the photomicrographs in Figure 3, TP-GFP expression in hMSCs was higher when using lentiviral vectors compared to non-viral vectors. Additionally, cells transfected with the viral vector maintained a healthier morphology than those treated with the PEI non-viral vector after 2 days of transfection. These observations indicate that polycationic PEI, at higher N/P ratios such as 5 and 7, was toxic to hMSCs, even though these ratios were safe for HEK293T cells, as shown in Figure 2. The optimal N/P ratios (around 5–7) of PEI/plasmid complexes for gene transfection of hMSCs are consistent with the published accounts [25]. Consequently, lentiviral vectors were chosen as the TP gene delivery method for hMSCs in subsequent studies.

**Figure 4:**
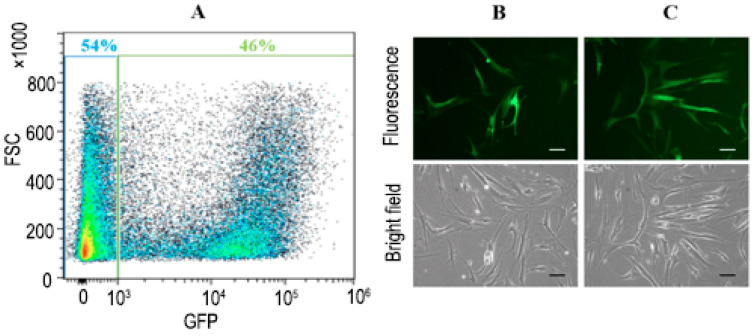
Cell sorting of hMSCs expressing TP-GFP. (**A**) A total of 46% of hMSCs with TP-GFP expression were sorted. Fluorescence and bright-field images of TP-GFP expression in hMSCs (**B**) before and (**C**) after cell sorting. The scale bar represents 100 µm.

The marker protein (GFP) displaying its characteristic green fluorescence in Figure 3 indicates that the heterologous TP protein was also correctly folded, as the expression of the two proteins is linked. Therefore, the level of TP protein expression in hMSCs can be inferred by the intensity of the green fluorescence observed in the cells [26]. The hMSCs expressing TP-GFP were further sorted from non-expressing cells based on GFP fluorescence, as shown in Figure 4. Figure 4A indicates that 46% of the total cell population was successfully infected with lentiviral vectors, exhibiting GFP expression. The sorting results confirmed that both the delivery efficiency and the protein expression level of TP-GFP in hMSCs using lentiviral vectors were much better than with non-viral PEI vectors (~15%). This relatively low (15–20%) hMSC transfection rate using PEI-based non-viral vectors has been reported previously [27]. As illustrated in Figure 4B,C, the percentage of post-sorting hMSCs expressing TP-GFP expression was increased to about 80%, greatly enhancing the potential for effective prodrug-to-drug conversion.

### 3.4. TP-GFP Protein Expression in hMSCs

**Figure 5:**
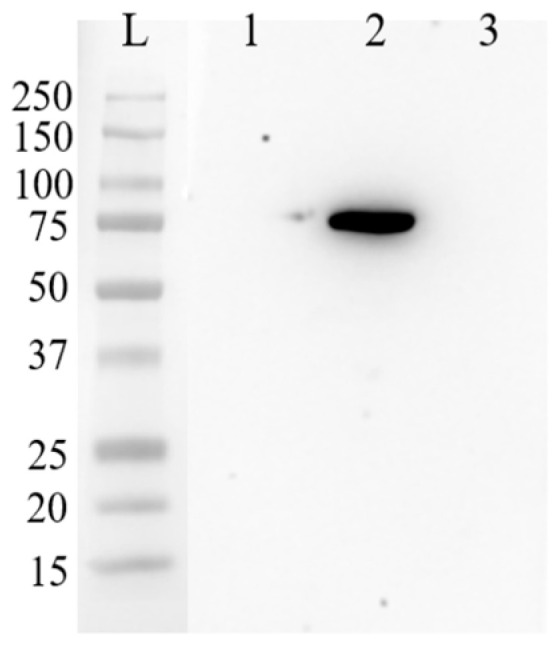
Western blot analysis of TP-GFP expression in hMSCs detected using a TP antibody. Lane L is the protein ladder. Lane 1 shows bands of TP-GFP protein detected in the culture medium of hMSCs following viral infection. Lane 2 displays bands of TP-GFP expression in the cell lysate of hMSCs delivered by lentiviral vectors. Lane 3 shows bands of TP-GFP protein detected in the cell lysate of hMSCs without viral gene delivery.

A Western blot assay was performed to verify the expression levels of exogenous TP and GFP proteins in hMSCs, using TP and GFP antibodies, as shown in Figure 5. The molecular weight of the exogenously expressed TP is approximately 55 kDa, that of GFP is around 25 kDa, and that of the TP-GFP fusion protein is ~80 kDa. In Figure 5, three samples from hMSCs and their culture medium were analyzed using a TP antibody. Lane L represents the protein ladder. Lanes 1 and 2 display the TP-GFP bands in the culture medium and the cell lysate from hMSCs infected with viral vectors. The absence of distinct bands in the culture medium suggests that the TP protein remained within the cells, which is crucial for the success of the GDEPT (gene-directed enzyme prodrug therapy). This ensures that TP remains inside the hMSCs while homing to tumor sites, preventing the TP protein from leaking throughout the body and triggering side effects from the conversion of doxifluridine to toxic 5-FU. Lane 3 shows the control hMSC sample without viral infection, indicating that there is no endogenous TP protein present in the hMSCs.

### 3.5. TP Enzyme Activity in hMSCs

**Figure 6:**
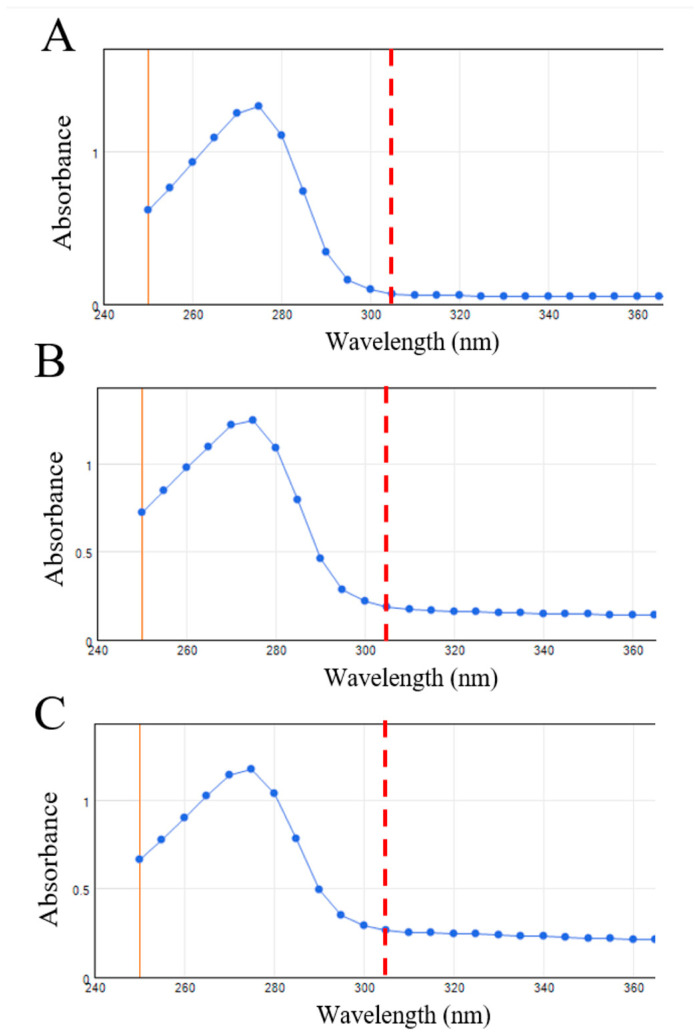
Enzyme activity of TP protein expression in hMSCs without TP gene delivery (**A**), with non-viral vector delivery (**B**), and with lentiviral vector delivery (**C**). The spectrometer reading at 305 nm increased from 0.06 (**A**) to 0.13 (**B**) and finally to 0.25 (**C**), correlating with the level of TP expression in the cells.

The enzyme activity of TP protein in hMSCs was measured using an enzyme activity assay, and the spectrometer readings are presented in Figure 6. The optical density (OD) reading at 305 nm reflects TP enzyme activity [7,28], with higher OD values indicating greater enzyme activity. All three samples contained the same number of cells and were treated with an equal amount of the prodrug doxifluridine. The varying OD readings suggest different levels of doxifluridine being converted to 5-FU by the TP protein expressed in hMSCs. As shown in Figure 6A, hMSCs without TP-GFP expression had an OD reading of 0.06, indicating no significant enzyme activity. This increased to 0.13 in cells expressing TP-GFP delivered via the PEI vector (Figure 6B), and further increased to 0.25 in cells using a viral vector for gene delivery (Figure 6C). These rising OD readings correspond to increased enzyme activity, which aligns with the observed green fluorescence and Western blot results aforementioned. It is noteworthy that the level of GFP expressed along with TP could potentially impact the absorbance reading; however, it has been reported that the absorbance of GFP at 305 nm is minimal (~0.01) [29]. Therefore, the expression level of GFP should not significantly impact the absorbance at 305 nm used in this study to assess TP enzyme activity.

### 3.6. Cell Viability of Co-Cultured hMSCs and A549 Cells Treated with Doxifluridine

**Figure 7:**
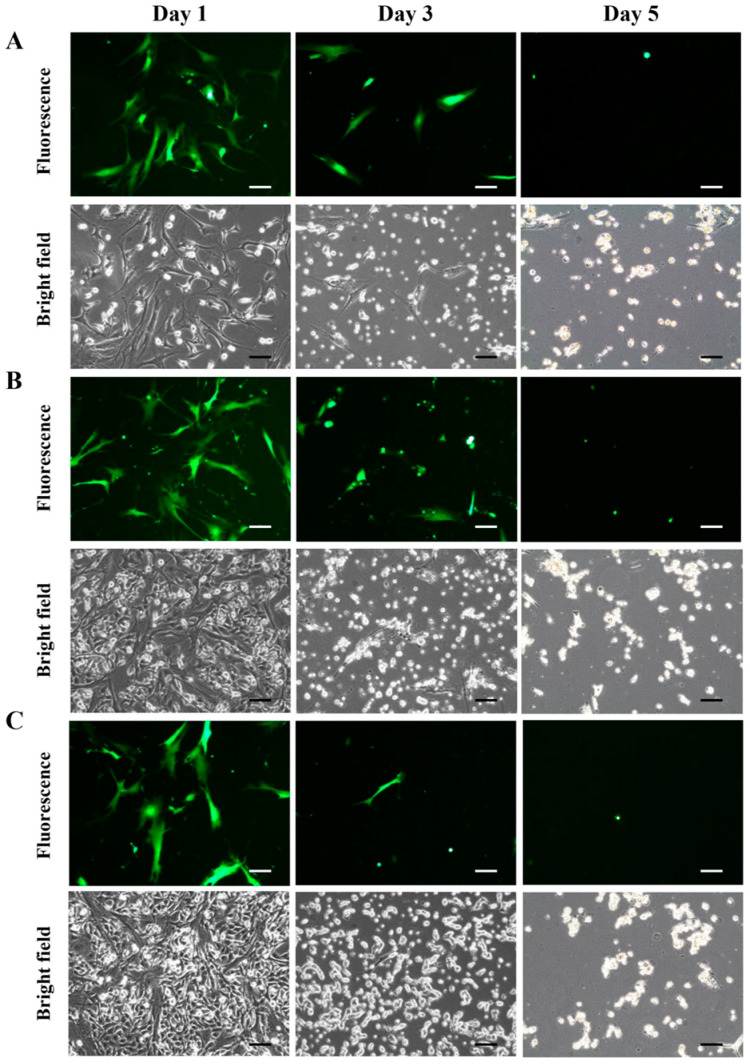
Cell viability of hMSCs and A549 cells co-cultured with prodrug doxifluridine. Fluorescence and bright-field images of TP-GFP expression in hMSCs co-cultured with A549 cells at the ratios of (**A**) 2:1, (**B**) 1:1, and (**C**) 1:2, following treatment with the prodrug doxifluridine on days 1, 3, and 5, respectively. The scale bar represents 100 µm.

Human mesenchymal stem cells (hMSCs) expressing exogenous TP-GFP were co-cultured with A549 lung cancer cells in varying ratios, maintaining a constant hMSC number, to assess cell viability following treatment with the prodrug doxifluridine. Figure 7 presents fluorescence and bright-field images of the co-cultured hMSCs expressing TP-GFP and A549 cells at three different ratios: 2:1 (A), 1:1 (B), and 1:2 (C). The cultures were observed for five days post-doxifluridine treatment, with results recorded on days 1, 3, and 5. In group A, both hMSCs and A549 cells ceased proliferation by day 2. By day 3, A549 cells began to shrink and detach, while some hMSCs retained a healthy appearance but showed signs of shrinkage by day 4. By day 5, over 80% of both cell types were dead (Figure 7A). In group B, the number of both hMSCs and A549 cells began to decrease by day 3, with more than 80% cell death observed by day 5, similar to group A (Figure 7B). Group C showed slightly prolonged survival of A549 cells compared to the other groups, with about 50% of A549 cells still alive on day 3. However, by day 5, more than 80% of the cells in this group were also dead (Figure 7C). Across all three groups, both hMSCs and A549 cells were reduced to less than 20% viability by day 5 following prodrug treatment. The results indicate that hMSCs expressing the TP enzyme, delivered via a viral vector, were able to convert doxifluridine into the chemotherapy drug 5-FU, effectively killing both A549 cancer cells and hMSCs which carry the TP enzyme via viral transfection. Although the timing of cell death initiation varied between groups, final cell viability was consistently reduced to less than 20% by day 5.

**Figure 8:**
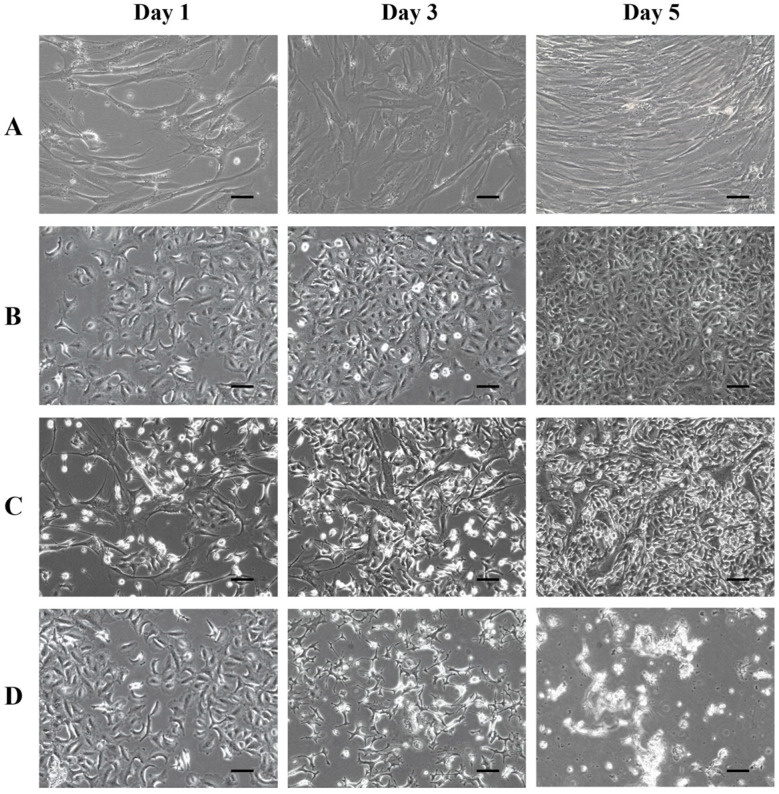
Verification of prodrug doxifluridine conversion to cytotoxic 5-FU by TP expressed in MSCs. Images of (**A**) hMSCs, (**B**) A549 cells, and (**C**) the co-culture of A549 cells and hMSCs treated with the prodrug doxifluridine on days 1, 3, and 5, respectively. Also shown are images of A549 cells (**D**) treated with the chemotherapy drug 5-FU on days 1, 3, and 5, respectively. The scale bar represents 100 µm.

To confirm that the conversion of doxifluridine to 5-FU was facilitated by the exogenously expressed TP protein in hMSCs, a control viability test was conducted by treating parental hMSCs and A549 cells with doxifluridine. Figure 8A–C show the cell viability over five days in cultures of parental hMSCs, A549 cells, and parental hMSC/A549 co-cultures following doxifluridine treatment. Neither parental hMSCs nor A549 cells exhibited cell death and continued to grow and reached 100% confluence over the five days at a similar rate to untreated cells (Figure 8A,B). Similarly, the parental hMSC/A549 co-cultures also showed no cell death and reached 100% confluence within the same period (Figure 8C). The morphology and viability of both parental hMSCs and A549 cells remained unaffected by doxifluridine. However, when treated with the chemotherapy drug 5-FU, A549 cells began to die within 24 h, and their viability steadily decreased to less than 10% confluence after five days (Figure 8D). This reduction in cell viability was similar to that observed in the co-cultures of A549 cells and hMSCs with exogenous TP expression treated with doxifluridine, confirming that the TP-expressing hMSCs successfully converted the prodrug into 5-FU, leading to cell death. These control results demonstrated that doxifluridine alone has no toxicity toward hMSCs, A549 cells, or their co-cultures, and both cell types maintained the same growth rate as untreated cells under prodrug treatment. It is worth noting that despite A549 cells, like other cancer cells, containing some levels of endogenous TP [30], the intracellular concentration might be too low; hence, there was no significant conversion of doxifluridine to 5-FU within the A549 cells. A549 cells treated with doxifluridine continued to grow and reached 100% confluence at a similar rate to untreated cells.

Taken altogether, the prodrug doxifluridine was first delivered into the cytosol of hMSCs and then converted into 5-FU by the exogenously expressed TP-GFP in hMSCs, which are transduced by lentiviral vectors. These hMSCs acted as vehicles for TP gene-directed enzyme therapy. Since no TP-GFP was detected in the cell culture media, it is presumed that hMSCs expressing TP are safe for healthy cells in vivo, as they home to tumor sites. Upon reaching the tumor, the toxic 5-FU would be released from the hMSCs, facilitated by the administration of the prodrug doxifluridine.

## 4. Conclusions

In this study, the TP gene was delivered into hMSCs using lentiviral vectors, which resulted in significantly higher protein expression compared to non-viral vectors. Within the hMSCs, the prodrug doxifluridine underwent extensive conversion to the chemotherapy drug 5-FU via the exogenous TP. The released 5-FU exhibited similar anticancer activity against A549 lung cancer cells to that observed with direct treatment using 5-FU. Overall, our findings suggest that the combination of the prodrug doxifluridine and the enzyme TP, delivered through hMSCs as carriers, has significant potential for use in gene-directed enzyme prodrug therapy (GDEPT) with minimal side effects.

## Data Availability

The data presented in this study are available on request from the corresponding author.
